# Analyzing Media Coverage of the Global Fund Diseases Compared with Lower Funded Diseases (Childhood Pneumonia, Diarrhea and Measles)

**DOI:** 10.1371/journal.pone.0020438

**Published:** 2011-06-08

**Authors:** David L. Hudacek, Shyama Kuruvilla, Nora Kim, Katherine Semrau, Donald Thea, Shamim Qazi, Andrew Pleasant, James Shanahan

**Affiliations:** 1 Center for Global Health and Development, Boston University, Boston, Massachusetts, United States of America; 2 World Health Organization, Geneva, Switzerland; 3 Canyon Ranch Institute, Tucson, Arizona, United States of America; 4 College of Communications, Boston University, Boston, Massachusetts, United States of America; Walter and Eliza Hall Institute of Medical Research, Australia

## Abstract

**Background:**

Pneumonia, diarrhea and measles are the leading causes of death in children worldwide, but have a disproportionately low share of international funding and media attention [1]–[3]. In comparison, AIDS, tuberculosis and malaria - diseases that also significantly affect children – receive considerably more funding and have relatively high media coverage. This study investigates the potential relationship between media agenda setting and funding levels in the context of the actual burden of disease.

**Methods:**

The news databases *Lexis Nexis*, *Factiva*, and *Google News Archive* were searched for the diseases AIDS, TB and Malaria and for lower funded pediatric diseases: childhood pneumonia, diarrhea, and measles. A sample of news articles across geographic regions was also analyzed using a qualitative narrative frame analysis of how the media stories were told.

**Results:**

There were significantly more articles addressing the Global Fund diseases compared to the lower funded pediatric diseases between 1981 and 2008 (1,344,150 versus 291,865 articles). There were also notable differences in the framing of media narratives: 1) There was a high proportion of articles with the primary purpose of raising awareness for AIDS, TB and malaria (46.2%) compared with only 17.9% of the pediatric disease articles. 2) Nearly two-thirds (61.5%) of the AIDS, tuberculosis and malaria articles used a human rights, legal or social justice frame, compared with 46.2% for the lower funded pediatric disease articles, which primarily used an ethical or moral frame.

**Conclusion:**

This study demonstrates that lower funded pediatric diseases are presented differently in the media, both quantitatively and qualitatively, than higher funded, higher profile diseases.

## Introduction

**Figure 1:**
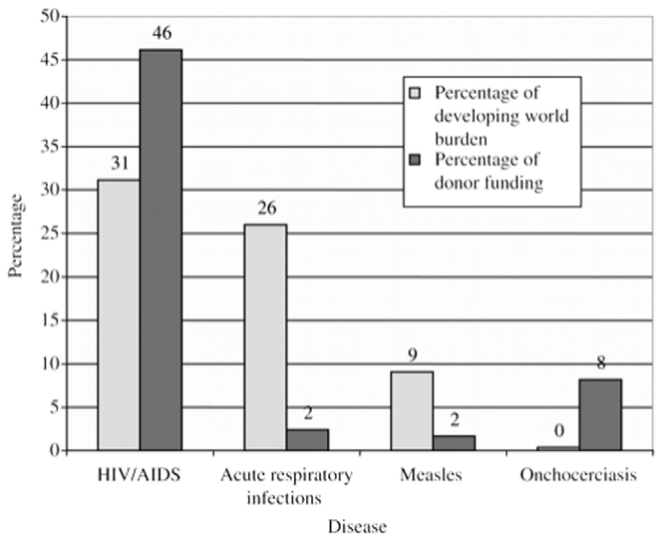
Percentage of Developing World Burden of Disease and Percentage of Donor Funding 1996–2003. **Summary**- Disparity between disease burden and donor funding. **Source- From** Shiffman J. Donor funding priorities for communicable disease control in the developing world. *Health Policy and Planning* 2006; 21(6); 411–420 by permission of Oxford University Press.

Clearly, variations in the burden of disease are well documented and many organizations and efforts have been developed in an attempt to respond to and lower the burden of disease on children. This is an area were the relationship between scientific data and social responses can be investigated. For instance, the substantial burden of disease from childhood pneumonia has been recognized but the disconnect between the disease burden and funding level for research and in development aid has been highlighted (Figure 1) [2], [3], [18]. Other diseases – in particular, AIDS, tuberculosis and malaria – have been in the spotlight of global health policy and funding. This focused funding has been necessary to mitigate the particularly devastating impact of AIDS in the developing world, especially in sub-Saharan Africa. AIDS-related programs have received important funding from such sources as the US President's Emergency Plan for AIDS Relief (PEPFAR). Malaria and tuberculosis received a boost in funding along with AIDS through the creation of The Global Fund To Fight AIDS, Tuberculosis and Malaria (GFATM). This study seeks to explore the relationship between one potential factor accounting for the prominence of some diseases on the world stage and the relative obscurity of others: the coverage and framing of articles in the mass news media.

Policy decisions about funding do not occur in a sociopolitical vacuum. The media play an important role in both providing information to policy makers as well as influencing public deliberation on public health issues [4], [5]. As an example, a media analysis on tobacco control suggests that neutral, fact-based, news reportage acts as a significant background for discussion, and that “active” media advocacy can shape public health policy [6], [7]. In HIV/AIDS, the dynamic nature of that health story over time - involving celebrities, sexuality, politics, and conflict - has played a role in shaping the policy agenda [8]. The mass news media is where the public often gets information about such public health topics; a 2003 survey by the Kaiser Family Foundation found that 72% of the U.S. public gets most of their information about HIV/AIDS from the popular media [8].

Previous media content analyses have examined the news coverage of neglected diseases like leishmaniasis and trypanosomiasis and high profile diseases like AIDS. However these studies have been conducted on specific topics rather than in comparative analyses [8], [9]. This study seeks to understand the media coverage of neglected pediatric diseases - childhood pneumonia, diarrhea and measles - as directly compared with policy and funding “success stories” such as AIDS, tuberculosis, and malaria. Understanding differences in media framing may influence the future work of activists and policy makers in shaping and promoting their policy agendas for neglected diseases like childhood pneumonia, diarrhea, and measles.

## Methods

The diseases of the GFATM - AIDS, tuberculosis and malaria - were chosen for comparison to neglected childhood diseases because of their high profile and substantial piece of total global health funding. While AIDS and tuberculosis and especially malaria affect children as well as adults, these diseases were singled out because of their success in attracting both funding and media attention. Diarrhea and childhood pneumonia were chosen as key pediatric diseases, given their tremendous burden of disease and disproportionate underfunding. Measles was also included in the study, as a childhood disease with a relatively low profile on the world stage, a proven low-cost intervention, and relative underfunding [10].

News articles for this media analysis were selected using the following online databases and search engines: *Google News Archive*, *Factiva* and *LexisNexis News*. Online news sources were utilized given their ease of access and the broad search capabilities of online search engines. A Project for Excellence in Journalism has also demonstrated a heavy emphasis of online news (as opposed to print and other media) on foreign news - particularly relevant given the global scope of this project [11].

There were two major categories of analysis: 1) a *Historical trend analysis* which involved a quantitative analysis of articles from 1981 to the present published on each of the diseases. The quantitative sample included all the articles available in online databases of print media - that is, online sources of newspapers as well as traditional print media articles available online; 2) a *Content and narrative analysis* of a sample of current online articles across geographical regions from January 1, 2008 to August 1, 2009 to understand the range of current sociopolitical dialogue around these diseases. This smaller sample allowed in-depth analysis of the selected media pieces: using a qualitative frame analysis to investigate *how* these stories are presented in the media. The sufficiency of qualitative sampling is determined by whether the thematic findings are repeated and predictable patterns emerge. If the initial sample had been insufficient based on these criteria, more articles would have been selected.

The search terms used in both the broad historic analysis and the geographic sample were: “childhood pneumonia”, “diarrhea/diarrhoea”, “measles”, “HIV, HIV/AIDS”, “TB/tuberculosis” and “malaria”. While these search terms are broad, they allowed for more standardized chronological comparison. The use of additional terms under the rubric of these overarching disease categories, such as rotavirus in addition to diarrhea, would have expanded the search, but cross-comparison across time and topic would be problematic given, for example, that advances specifically related to the rotavirus vaccine are relatively recent. For each disease category, the search terms “child”, “children”, and “pediatric” was added to generate a list of articles relating to a pediatric population. A single reference to these pediatric population terms was the minimum requirement for such articles to be included as mentioning or referencing children in the quantitative analysis. Whether the articles were primarily focused on such pediatric populations was assessed in the qualitative frame analysis of the articles.

### Historical Analysis

To determine trends in the number of media articles over time covering the lower funded pediatric diseases as compared to AIDS, tuberculosis and malaria, the search engine *Google News Archive* was used with the disease-specific search terms. These terms were searched from the period of 1981- the emergence of HIV/AIDS - to December 31, 2008. To clarify whether the disease terms were a prominent focus of the article, rather than just appearing once (possibly incidentally), a search for the terms in the headline or the first paragraph was done using the search engine *Factiva*. Total direct funding of these diseases from 1996–2003 (for which data are available) was compared to the number of articles as well as disease burden as measured by disability adjusted life years (DALYs), to analyze any possible correlation between funding, media coverage, and burden of disease.

### Content and Narrative Analysis

#### Media Sampling Strategy

To choose global media with the broadest reach and greatest prominence, the top circulating newspapers in each of the World Health Organization (WHO)'s six geographic areas were sampled and coded by geographical region. [The six geographical regions used in coding are: WHO African Region, WHO Region of the Americas, WHO South-East Asia Region, WHO European Region, WHO Eastern Mediterranean Region and WHO Western Pacific Region.]

Papers with leading circulations in these regions, and their online English language editions, were identified based on World Association of Newspapers listings. For these leading papers, the top hits on the search engines for the search terms (e.g. “childhood pneumonia”) were used [12]. If high circulation papers did not have articles for the search terms, then the top hits for those disease terms from other news sources in that region were used. Within each geographic region where several countries might have a prominent global media presence, countries with the highest prevalence of childhood pneumonia, diarrhea and measles based on WHO data (e.g. India, China and Pakistan) were targeted [2].

Sampling from the six WHO geographic regions was meant to help account for varying socio-cultural and geographic factors in media coverage (maximum variation sampling). With six diseases (AIDS, tuberculosis, malaria, childhood pneumonia, diarrhea and measles) being compared, a sample of three articles of each of the six diseases from the top circulating papers of each of the WHO regions were screened, for a total of 108 articles targeted for inclusion.

#### Article Inclusion Criteria

Articles were selected based on inclusion of the disease-specific search terms in the full-text. These terms had to be mentioned at least twice in the article to be included in that category of health topic. Articles were searched using the online search engines for the time period from January 1, 2008 to August 1, 2009. 78 news articles met the inclusion criteria. Of these, 39 met inclusion criteria for childhood pneumonia, diarrhea or measles (lower funded group), and 39 met inclusion criteria for AIDS, tuberculosis or malaria (higher funded group).

#### Coding Framework – quantitative and qualitative

Both quantitative content analysis and qualitative frame analysis were employed. The full coding framework is available in the Supporting Information S1. Three coders were trained in using the coding sheet. Data were coded and analyzed using HyperResearch software (Qualitative Analysis Tool Software, Version 2.8.2). Key elements of the coding framework are summarized below.

#### Quantitative content analysis

The articles selected for analysis, in to being categorized by the six diseases being compared, were also coded by: type (e.g., op-ed); age of affected population; and media ownership.

#### Qualitative analysis

Frame analysis takes into account *how* issues are presented in the media. Such analysis is crucial to understanding meanings and effects on audiences [13]. Narratology, or understanding the framing of narrative, is one method of frame analysis [13]. Studies suggest that news media are searched by readers for certain kinds of story structure, and these structures of stories are remarkably similar across cultures; a strong narrative is a vital and common way through which audiences make sense of presented information [14]. Thus the coding framework incorporated an analysis of the narrative elements of the news articles, including tone, meta-story, protagonists, antagonists and resolution.

In addition, because perceptions of economic costs may influence public policy, articles were analyzed for costs mentioned. Given that misperceptions of the efficacy of proven interventions may cause underfunding, articles were also analyzed for interventions mentioned as well as their reported impact.

#### Reliability

Three coders coded the selected articles using the coding sheet. Inter-rater reliability was tested for agreement by coders cross-coding the same articles and comparing percent category agreement. Using an iterative process, intercoder reliability achieved a reliability of .72. That is, an initial sample of articles was coded as a test of the coding framework. Initial intercoder reliability was .51; further revision of the coding framework, training of coders, and review to achieve consensus on framing analysis led to a reliability of 0.72, which is considered acceptable for exploratory studies such as this one.

### Generalizability

#### Quantitative analysis

Statistical analysis of categories with significant differences was performed using a chi-square (X^2^) test.

#### Qualitative analysis

The qualitative findings were analyzed in the context of “agenda setting” and generalizabilty established with reference to related theory, where agenda setting has three components: *framing*, *schema*, and *priming* [15]. Analysis of framing focused on narrative elements. Schema refers to how people might organize their thinking based on exposure to media elements, and in this study was analyzed by examining the “meta-story”, i.e., the overriding narrative of these diseases in the media. Priming refers to how these elements are mobilized in the public sphere by the frequency and prominence of these stories, which in our study was examined in the more quantitative historic analysis of the number of articles on each disease and story prominence.

## Results

**Figure 2:**
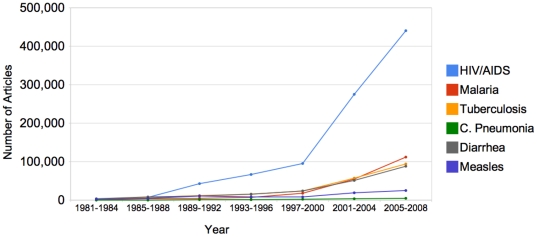
Number of articles listed on Google News Archive for Childhood Pneumonia, Measles, Diarrhea, HIV/AIDS, Malaria and Tuberculosis from 1981–2008. **Summary**- Increase in number of articles over time for selected disease topics. **Source**- *Google News Archive* accessed 9/28/09.

In the historical analysis, between 1981 and 2008 there was a significantly greater number of articles covering or referring to AIDS, tuberculosis and malaria than to childhood pneumonia, diarrhea and measles (Figure 2). Combined, there have been 1,344,150 articles about, or mentioning, AIDS, TB and malaria, while there were only 13,600 articles about, or mentioning childhood pneumonia, 83,900 on measles and 166,000 on diarrhea (a total of 291,865). Notable is the marked increase in media coverage for AIDS, TB, and Malaria after the year 2000 with the Millennium Declaration and the formation of the Global Fund. This is most marked for HIV/AIDS. There is also a similar but less marked trend for articles covering malaria, tuberculosis, and diarrhea.

**Figure 3:**
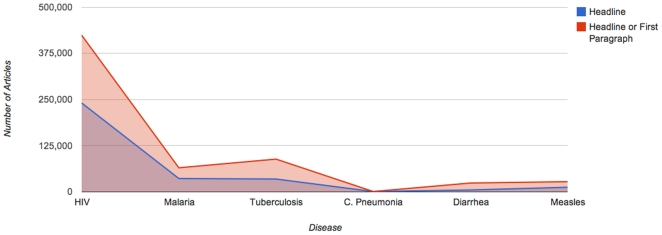
Number of Articles Featuring Disease in Headline or First Paragraph 1981–2008. **Summary**- Greater number of articles focused on global fund diseases as compared to lower funded diseases. **Source**- *Factiva*, accessed 10/26/09.

To determine whether the diseases were the primary or prominent focus of the news articles, online media were analyzed from 1981–2008 for the search terms appearing in the headline or first paragraph. There was a ten-fold difference in the number of articles prominently featuring AIDS, tuberculosis or malaria (576,298 articles) as compared to childhood pneumonia, diarrhea or measles (50,181 articles) (Figure 3).

While the total number of articles mentioning diarrhea are comparable to the number of articles on malaria or tuberculosis (Figure 2), the number of articles focused on diarrhea - and specifically childhood diarrhea - is much less (Figure 3).

**Figure 4:**
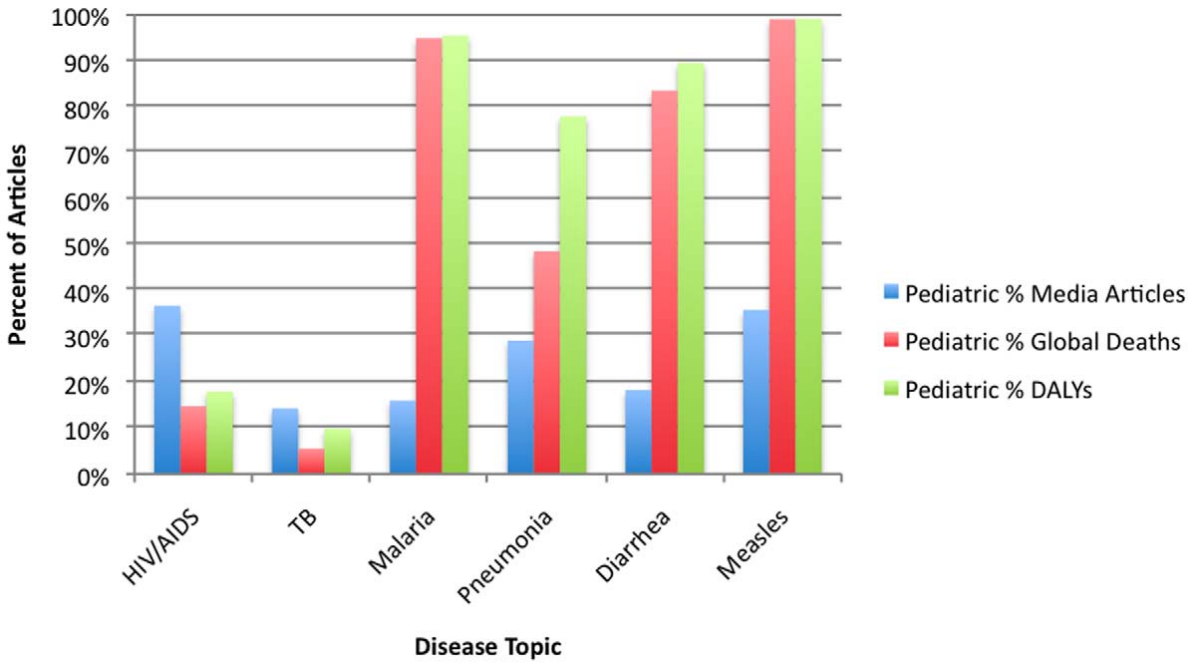
Comparison of the Percent of Articles 1981–2008 Referencing Pediatric Populations, Pediatric Percent of Global Deaths, and Pediatric Percent of Global Burden of Disease. **Summary**- Relative increased percentage of pediatric AIDS and TB articles as compared to pediatric disease burden for those diseases. **Sources**- *Google News Archive* accessed 4/14/10; WHO Global Burden of Disease Report 2004 update [http://www.who.int/healthinfo/global_burden_disease/GBD_report_2004update_full.pdf].

For each of the diseases searched, there is a large discrepancy between the amount of pediatric news coverage as a percentage of each disease's total news coverage, and the relative disease burden the pediatric population carries (measured by DALYs) (Figure 4).

Each of the diseases where the pediatric population carries the greatest burden - malaria, pneumonia, diarrhea and measles, were underrepresented in the news media. The two diseases where the pediatric population carries the minority of the burden - HIV/AIDS and tuberculosis - were, if anything, “over-represented” in the media. This was most marked for HIV/AIDS, where the percent of news coverage for the pediatric population was double the pediatric disease burden percentage, as measured by DALYs (36.6% of the AIDS news coverage versus 18.0% of the disease burden).

**Figure 5:**
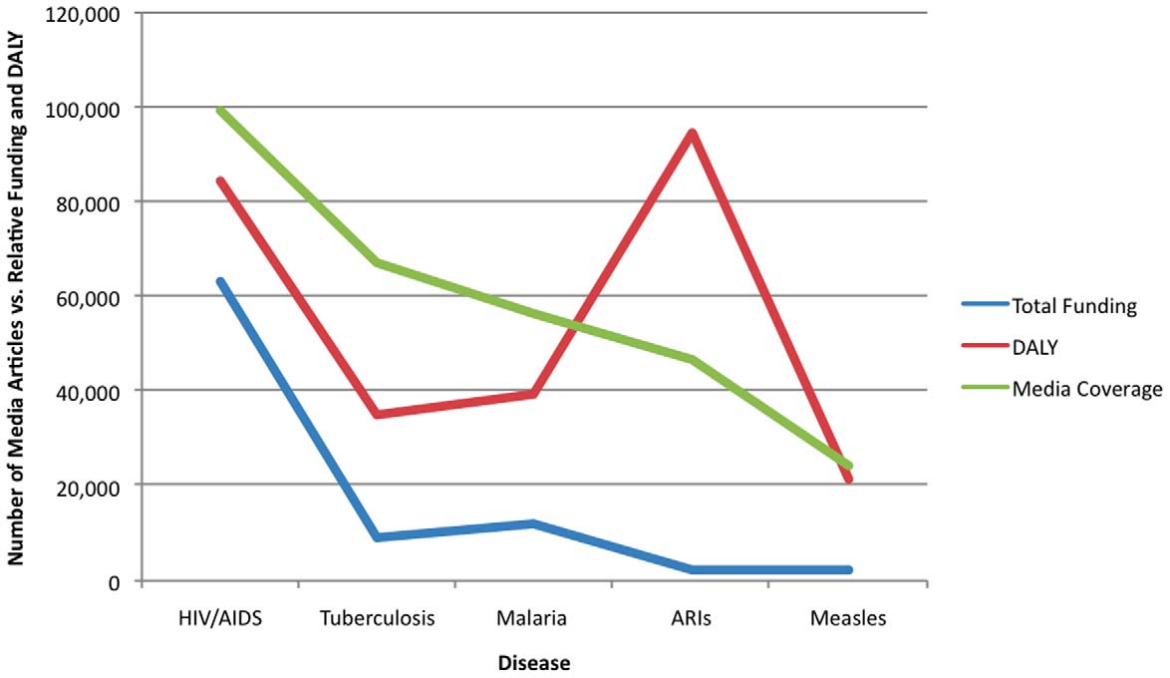
Comparison of Funding (1996–2003), Media Coverage and Global Burden of Disease. **Summary**- Disparity between disease burden of acute respiratory infections (including childhood pneumonia) and media coverage and funding. **Key**- Media coverage in absolute numbers of articles 2002; DALYs in thousands; Funding amounts in hundreds US Dollars; ARIs = Acute Respiratory Illnesses. **Sources** - *Google News Archive* accessed 10/20/09; Shiffman J. Donor funding priorities for communicable disease control in the developing world. *Health Policy and Planning* 2006; 21(6);411–420.

When media coverage for each of the six diseases is compared to both disease burden and funding, there appears to be a correlation between media coverage and amount of funding rather than disease burden (Figure 5). [Similar data for funding were not available for diarrhea] [16].

In the quantitative content analysis the general underrepresentation of pediatric populations was highlighted in the 78 articles sampled. While childhood pneumonia and measles were obviously focused on children under the age of five, there was little focus among the AIDS, tuberculosis and malaria articles in terms of pediatrics. Sixty-six percent of the AIDS, TB and malaria articles covered all age groups or specifically adults, while only 2 (5.1%) of the articles in this group focused on children [p<0.001] (Table 1).

**Table 1:** Coding Results for 78 Sample Cases (HyperResearch coding output) *n* = 78. (Childhood Pneumonia, Measles, Diarrhea, AIDS, Tuberculosis and Malaria).

	Lower Funded Diseases			Highly Funded Diseases	
		*Number of Articles (n = 39)*	*%*	*Number of Articles (n = 39)*	*%*
**Story Tone**	Negative	13	33.3%	11	28.2%
	Neutral	13	33.3%	14	35.9%
	Positive	10	25.6%	14	35.9%
**Title**	Negative	17	43.6%	19	48.7%
	Neutral	12	30.8%	12	30.8%
	Positive	11	28.2%	11	28.2%
**Resolution**	Likely	14	35.9%	14	35.9%
	Not Likely	3	7.7%	7	17.9%
	Ambiguous	8	20.5%	2	5.1%
**Story Details**	Broad Statistics	32	82.15%	26	66.7%
	Global Context	17	43.6%	22	56.4%
	National Context	23	59.0%	18	46.2%
	Biologic Agent	15	38.5%	10	25.6%
**Story Action**	New Initiative	9	23.1%	15	38.5%
**Plea to Audience**	Yes	18	46.2%	16	41.0%
	Explicit	10	25.6%	4	10.3%
	Implicit	8	20.5%	12	30.8%
**Population Focus**	Pediatric	36	92.3%	2	5.1%
	Adolescent	4	10.3%	3	7.7%
	Adult or all	6	15.4%	20	51.3%
**Purpose of Article**	Raise Awareness	7	17.9%	18	46.2%
	New Discovery	10	25.6%	8	20.5%
**Theme of Sub-Theme**	Global Movement or Human Rights	18	46.2%	24	61.5%
	Moral/“Right thing to do”	19	48.7%	11	28.2%

Other quantitative content analysis showed no significant differences between the pediatric diseases and the AIDS, tuberculosis and malaria group of articles in regard to the article type (i.e., health section, op-ed, or primary news story) and whether the news source was publicly or privately owned. In both groups there was a strong trend towards news sources with mixed ownership (i.e., combined public and private ownership).

In the qualitative narrative frame analysis of the news stories as a whole, there was an even distribution of tone in terms of negativity, neutrality and positivity (Table 1). Twice as many stories that suggested a resolution to the health problem framed it as likely, as compared to unlikely. The most common story dimension was broad statistical data. The most common story action focused on a new initiative. Similarly, the most common broader story context, or meta-story, focused on new policies. Collectively, the articles were evenly split between framing the story in a global context and a national context. Only 12% of the articles portrayed the health topic as an atypical disease, and a significant proportion posed the disease as a risk to the audience. A majority made some type of plea, either implicit or explicit, to the reading audience.

There was a higher proportion of articles with the purpose of raising awareness in the AIDS, TB and malaria group (46.2%) than articles on childhood pneumonia, diarrhea and measles (18.6%).

Economic costs and interventions mentioned in the articles were part of the coding framework for analysis, but not enough instances were noted in the articles for any meaningful comparison.

The differences in the narrative analysis between the lower funded pediatric group when compared to AIDS, tuberculosis and malaria, suggest differences in the meta-stories/sub-themes and the protagonists (Table 1). Sixty-one percent of the highly funded disease articles used human rights or social justice as the major theme (meta-story) or sub-theme, while less than half (46.2%) of the lower funded disease articles used these as a major theme or sub-theme. Two-thirds (66.7%) of the AIDS, tuberculosis and malaria articles had a clearly identified protagonist, such as a celebrity, activist, or organization. Only 44% of the childhood pneumonia, diarrhea and measles articles identified such a protagonist.

## Discussion

This study found that while there are similarities in the presentation of global health news stories, there are important differences between the quantity and the qualitative framing of stories for highly funded global infectious diseases as compared to lower funded primarily pediatric diseases, which have as much or greater burden of disease.

Similarities in media coverage across all the topics include the types of news sources, the newspaper sections where these stories appear, and the general tone of these stories. Most of the news stories included broad statistical data, usually suggesting potential risk of these diseases to the audience, and frequently in a national context. These stories often revolve around a new initiative or plan, with either an implicit or explicit plea to the audience for these initiatives, and are more likely to suggest a resolution to the problem than not. This hopefulness is an interesting contrast to the tone of the articles, which as a whole are more likely to be negative than positive.

There are also key differences between coverage of the different diseases. The most glaring is the sheer volume of news coverage about the diseases of The Global Fund To Fight AIDS, Tuberculosis and Malaria (GFATM), which dwarf the number of stories about the disease with the greatest burden: childhood pneumonia. Measles was also underrepresented, but not to the same degree. Of interest is the relatively high number of articles mentioning diarrhea overall, but this could be related to adult diarrhea associated with travel and tourism. When headlines and first paragraphs are searched for diarrhea, the number of articles actually focusing on childhood diarrhea is relatively small, and more closely mirrors the other lower funded pediatric diseases. Furthermore, when the number of articles for diarrhea is disaggregated for infant and other pediatric populations, this paucity of news coverage is even more pronounced.

News stories on AIDS, tuberculosis and malaria also tend to have a higher profile protagonist (e.g. activist, celebrity or prominent organization such as Doctors Without Borders) working as part of a global health or social justice movement, and a more global story dimension. A Kaiser Family Foundation study of media coverage of HIV/AIDS from 1981–2002 bears out this shift in focus from domestic disease to a global perspective, at least in terms of the AIDS pandemic [8]. In contrast, stories about the lower funded diseases have a lower profile protagonist (if identified at all), usually as part of a national program, and are less likely to have a broader meta-story that ties into a world movement. A significant proportion of these pediatric articles focus on vaccinations. Another important difference was a greater focus on raising awareness in the AIDS, TB and malaria articles.

A striking finding is how little focus there is on children in the media covering diseases that afflict mostly pediatric populations. Of interest, and the most glaring example, is malaria, where 95.5% of the disease burden is carried by children, but where focus on the pediatric population accounts for only 16.0% of the media coverage [17]. And this is for a high profile disease, as measured by total news coverage and by funding. Also of interest is how HIV/AIDS once again shows its exceptional nature - here the pediatric news coverage is double the actual pediatric burden of disease. This over-representation is also seen, to a lesser extent, with tuberculosis. Pediatric tuberculosis has traditionally received less attention in public policy because of the lower infectivity of children with tuberculosis as compared to adults. [18] There are a number of important points in this context. First, the media does not focus on pediatric populations - this may be because children are a relatively voiceless population. Second, such media representation may not be enough; even within the world of HIV/AIDS, children are vastly underserved in terms of receiving anti-retrovirals as compared to adults in developing countries [19]. Finally, even if pediatric populations are underrepresented in the media, there may be ways of compensating in terms of achieving funding - that is, through attachment to a high profile fund like the Global Fund to Fight AIDS, Tuberculosis and Malaria. This has clearly worked for a Global Fund disease that significantly affects a pediatric population - malaria.

There are a number of possible explanations for the quantitative differences observed in this study. There is a strong trend for an increase in the number of “hits” using *Google News Archive* over time for all of the diseases searched. This may be due to the rise of the internet over this time period, with more venues for news stories, as well as more uploading of material over time. This could also be due to the already observed international bent of online media - with the increase in online news sources more international coverage leads to more stories about diseases with a global impact. Could this increase also be due to a general increase in funding for these diseases overall? In particular, the advent of the Global Fund for AIDS, Tuberculosis and Malaria (GFATM), the President's Emergency Plan for AIDS Relief (PEPFAR), and the Bill and Melinda Gates Foundation could have played a role in this trend in funding and subsequent increase in media coverage for the higher funded diseases. The rise in the number of HIV/AIDS articles around 2000 is most striking, and this study likely underestimates the number of articles referencing AIDS [given the search algorithm for *Google News Archive* does not distinguish between the word “aids” as in helps, and “AIDS”, the disease, only the narrower HIV or HIV/AIDS search terms were used]. Malaria and tuberculosis hew to this rising curve of AIDS articles over time most closely, and the possibility of a “coat-tail” effect should be considered. Specifically, the lumping together of these higher funded diseases in the GFATM may have broadened not only their funding, but media coverage as well through the announcements of new initiatives and policies engendered by this money.

The qualitative differences in this study also raise interesting questions. This study observes trends towards a human rights movement and social justice framework within the AIDS, tuberculosis and malaria articles, as well as a trend towards raising awareness. This may be led by the inherent nature of the AIDS epidemic, with all of the issues of gender and sexual preference discrimination, as well as the mobilization of previously disenfranchised groups playing a role. AIDS activism has historically utilized a human rights framework to great effect. When the data in this study are disaggregated, the malaria and tuberculosis articles seem to hold to this activist/global movement framework, but this, again, may be related to the grouping of these diseases in the GFATM. Likewise, the moral, “right thing to do” framework of the lower funded disease articles may be due to the inherent nature of these primarily pediatric diseases, where there is no real child activist movement.

Examples of these types of thematic analyses reveal the challenges inherent in qualitative analysis. Some articles were relatively easy to classify. For example, the following is the opening paragraph categorized as a human rights/legal/social justice meta-story: “HIV/AIDS high-risk groups, who are already reluctant to seek medical help for reproductive health services for fear of stigma, risk greater discrimination if the state includes religious values in articles regulating reproductive health services, a doctor warns.” [20] From an opinion piece titled ‘Pneumonia: Indonesia's forgotten child killer’, the “right thing to do” framing seems clear: “And beyond increasing knowledge, I hope that those in power take urgent steps to reduce this disease's impact. Let's renew our focus on Indonesia's promising future by investing in those who will drive it – our children.” [21] A more dubious example reveals the perils of such analysis. From an article titled ‘IMF funding ‘fuelling TB deaths’ the following could be interpreted in several frames: “Strict conditions on international loans have been blamed for thousands of extra tuberculosis deaths in eastern Europe, and former Soviet republics. Analysts from Cambridge and Yale universities said they had led to less being spent on healthcare.

As a result TB in countries with International Monetary Fund loans rose sharply, they claimed.” [22] An additional quote of David Stuckler from Cambridge University in the same article goes on to say “If we really want to create sustainable economic growth, we need first to ensure that we have taken care of people's most basic health needs.” [22] Collectively the framing in this article could be described as economic, social justice, or “right thing to do”. In practice, the frame analysis of this study allowed for multiple codings in the same article, so this article was coded as using multiple frames.

Other qualitative aspects of the nature of these diseases - not answered in this study - could affect media coverage. For example, is the “simpler” nature of a disease such as malaria, with only one pathogen, and relatively straightforward prevention (bed-nets) and treatment (a few days of pills) related to its greater success in attracting media attention and funding? In addition, is there nothing new, and by implication, newsworthy, in the world of childhood pneumonia, diarrhea, and measles? Prevention and treatments are known, but there are relatively few initiatives, especially in comparison with the higher funded diseases like AIDS, where people are marching in the streets, newly funded programs are constantly be announced, and exciting developments like a possible AIDS vaccine always seem to be on the horizon. For a story to “have legs”, it has to move. Lastly, the power of celebrity involvement in the funding and attention for higher profile, better funded diseases should not be underestimated.

### Implications for policy and practice

**Figure 6:**
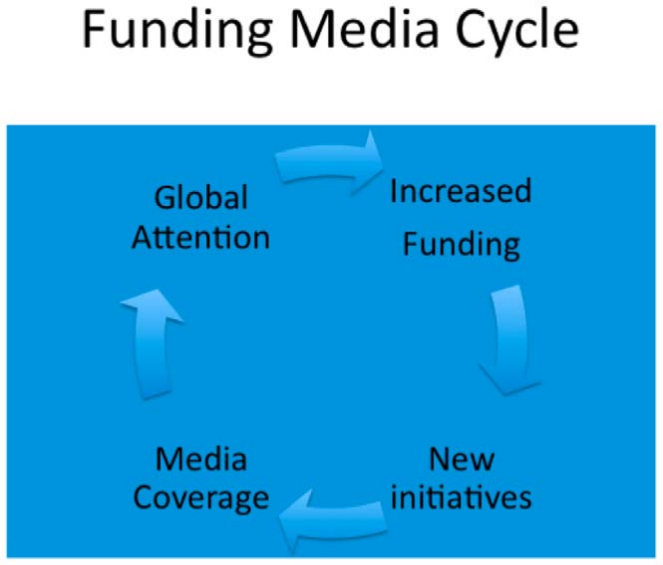
Funding Media Cycle. Proposed Relationship Between Media Coverage and Disease Funding.

This quantitative analysis raises the question: is there a cyclical relationship between global funding and media coverage (Figure 6)? Activism and advocacy injected into different stages of this cycle could influence both funding and media coverage. While it would be difficult for any study to tease out the relationship between the two (correlation does not mean causation), the degree of correlation is not negligible. It is also clear from the media reviewed that a proportion of the articles are reports of new initiatives that are generated by more funding, consistent with such a cyclical relationship.

The trends suggested in the qualitative analysis raise questions for activists and policymakers in terms of framing. Which framework is advantageous: a “moral/right thing to do” or a “legal”, “human rights” framework? There are examples where both moral and human rights or legal frameworks can be successful. The “moral/right thing to do” framework can be a potentially powerful way to change public policy. A notable example affecting pediatric populations is the Ottawa Treaty to ban antipersonnel landmines [15]. NGOs were very effective in using intense media coverage by: 1) *framing* this as a moral issue 2) putting a potentially complicated topic into a relatively straightforward *schema* for people to be able to process the information and 3) *priming*, that is, activating these frames in the public sphere through active campaigns and subsequent broad media coverage. A prominent tool in this media campaign was the testimony of landmine victims. But the “moral/right thing to do” framing has appeared to have had little success in generating either significant funding or media attention for certain neglected diseases. Perhaps this is because there are no real childhood pneumonia, diarrhea or measles victims to testify - they either succumb to the disease or get better.

Evidence for a legal, human rights approach to a pediatric disease is suggested by the success of the suit brought by the media savvy Treatment Action Campaign (TAC) in South Africa to change governmental policy on providing nevirapine to reduce mother to child transmission of HIV [23]. While this was primarily a legal approach, and not a media policy approach, the success of this activist organization is notable. In settling the suit in TAC's favor, the Constitutional Court specifically cited the constitutional rights of the children to “basic health care services.” Again, this approach might seem, on the surface, to be steeped in the nature of HIV/AIDS. But the ruling is not based in the specifics of HIV, but rather in the right to health, particularly for children. The benefit of such an approach to gain treatment and access for children is access to a legal framework that already exists in some countries (namely the International Covenant on Economic, Social and Cultural Rights and also the Convention on the Rights of the Child) but is explicit in few national constitutions.

Whether a human rights approach for pediatric populations can be broadened from a purely legal approach to a media and funding framework remains to be seen. But our analysis suggests there may be much to learn for policymakers from the history of such successes, warranting further study. A dichotomy does not need to exist between those seeking childhood pneumonia funding and those seeking funding for AIDS and other diseases. Rather, the success of agenda setting by the AIDS community may be duplicated in other contexts.

There are a number of limitations to this study. First, the time frame for the articles in the content analysis sample was narrow, from January 1, 2008 to August 1, 2009. This sample looked at the most recent news cycle; media coverage about a topic such as AIDS is constantly in flux, and evolves over time [8]. If funding for major initiatives like the Global Fund for AIDS, tuberculosis and malaria were affected by the media, or vice versa, it may also have been affected by media coverage over the past decade or more, not the most recent news cycle. [In this study the longer time frame of the historic quantitative analysis bears out important differences in the number of articles before these funds were created.] In addition, the search terms, such as “HIV/AIDS” and “diarrhea”, are broad; the use of additional terminology would have made the search more targeted. Other limitations are the lesser number of articles on childhood pneumonia, diarrhea and measles in some of the geographic areas studied, the grouping of different diseases into two comparison groups, and limitation to English language only online news sources. A future area of study could include television, film and radio. Finally, internet search databases such as *Google News Archive* are unstable as tools and depend on the proprietary algorithms used that are continually changed; thus results of searches for a defined time period can differ over time. Relative numbers are thus more pertinent than absolute numbers.

For the qualitative frame analysis, this was a preliminary analysis of a limited number of articles that highlighted certain themes. More research is needed to investigate the trends suggested in these findings.

### Conclusion and recommendations

The preliminary media analysis in this study raises a number of questions for those seeking funding and media attention for neglected pediatric populations. Should activists re-think the framing of neglected childhood diseases in the context of agenda setting for media, such as a legal, human rights or social justice framework? Should activists be engaged from the ground up (possibly the parents of children lost to these diseases, children affected by the diseases, and community activists such as community health workers, might project a proactive, dynamic story)? Finally, does it make sense to expand the GFATM to include neglected diseases, based on global burden of disease and other agreed on criteria by the global public health community? This would be a way to take advantage of the “coat-tail” effect of grouping diseases with AIDS and avoid separate funding organizations when effective infrastructure and administration already exist. A notable example suggested by this study is malaria - a primarily pediatric disease with significant funding from the GFATM.

Clearly, more research in this area is needed. Directions for future media research could include examining qualitative (especially narrative) trends over time, expanding the comparative approach of this study to other public health issues, and expanding the sample size of articles to test the theories established through qualitative analysis in quantitative studies.

The media are just one potential factor in how global health policy agendas are set and funds are appropriated to fight diseases. Many other factors, beyond the scope of this study come into play. The question of whether the media merely reflect or affect global policy is also beyond the scope of this study. But this study does demonstrate quantitative and possibly important qualitative differences between highly funded “media success” diseases like AIDS, tuberculosis and malaria versus the lower funded primarily pediatric diseases childhood pneumonia, diarrhea and measles. Broad, often tragic statistics, pleas to the audience, and new discoveries and initiatives are common themes to all these news stories. To garner the world's attention like the AIDS story, a story needs a strong narrative and an identifiable voice for those that have had less presence in the media.

## Supporting Information

Supporting Information S1 Media analysis coding sheet.
(DOC)
