# Supplementary material for: Analyzing Media Coverage of the Global Fund Diseases Compared with Lower Funded Diseases (Childhood Pneumonia, Diarrhea and Measles)
Source: PLoS One. 2011 Jun 8;6(6):e20438. doi: 10.1371/journal.pone.0020438 (PMC3110621; doi:10.1371/journal.pone.0020438)
Supplement: Supporting Information S1 — Media analysis coding sheet. (DOC) [file pone.0020438.s001.doc]

**Supporting Information S1-**

**MEDIA ANALYSIS CODING SHEET**

Article ID: ___________

Coder name: ___________

**1. News source:**

1. Name of news source (e.g. New York times, BBC online) ___________
2. Search Engine/Database Used (Factiva or Lexis Nexis)___________
3. Date accessed for analysis___________
4. Date article published ______________

**2. Category of News Source:**

a) News source ownership-

- Public Broadcast media (eg NPR) ___________
- Private media___________
- Mixed media (e.g. BBC)___________
- State owned (e.g. People’s Daily)___________
- Other ___________

b) If translated, original language of publication ___________

**3. Article 1:**

a) Health topic-

- Childhood Pneumonia _________
- AIDS _________
- TB __________
- Malaria __________
- Measles __________
- Other ____________

b) Word count___________

c) Headline (list in full) _________________

- Positive (triumph) _______
- Negative (tragedy) ________
- Neutral _________

d) Article type-

- Primary news story ________
- Health section story______
- Science or technology story_________
- Op-Ed____________
- Letter to the editor__________
- Other___________

e) Photo or graphic(s) total number ____________

Photo 1 (describe): ___________

- Positive (triumph) _______
- Negative (tragedy) ________
- Neutral _________

Photo 2 (describe): ___________

- Positive (triumph) _______
- Negative (tragedy) ________
- Neutral _________

f) Author and position, if listed (e.g. name and science reporter)_____________________

g) Other cited source (e.g., wire service: AP, Reuters; organizational press release etc) _______________

h) Purpose of article-

- Personal Health Info__________
- Community Health Info___________
- Emergency Alert___________
- New Discovery___________
- Neglected/Raising Awareness Disease__________
- New Policy__________
- Other___________

i) Population/s affected age-

- Child <5_________
- Child >5_________
- Adolescent 13-18 _______
- Adult>18 ________
- Elderly >65 ________
- All ages ________

j) Geographical focus:

- Specific country/ies ___________
- Specific region/s (e.g. WHO regional categories)___________
- Cross-border ___________

k) Pneumonia interventions mentioned-

- Reduction of indoor air pollution ________
  - Liquid fuel stoves _________
  - Improved solid fuel stoves ________
- Nutritional __________
  - Breastfeeding promotion __________
  - Zinc supplementation ___________
- Immunization ____________
  - Pneumococcal conjugate __________
  - Haemophilus influenzae type B __________
- Pneumonia case management __________
  - Community-based _________
  - Facility-based ___________
- Other _________

l) Costs mentioned -

**Cost of providing the health intervention**

- Administration (*overhead costs including space, furniture, equipment, utilities, maintenance etc*) ___________
- Planning (*per diem allowances, transportation, supplies, consultant fee etc)________*
- Training (*specific to intervention, e.g., training to use equipment or administer vaccine etc*) _____________
- Media and IEC (*designing messages, testing, revision and retesting, printing, radio or TV air time*) _______________
- Monitoring and supervision (*per diem allowances, travel allowances, personnel salaries related to supervision*) _____________
- Social mobilization (*All advertising and promotion activities, seminars etc*) __________
- Other _________

**Costs of accessing health intervention**

- Transportation (*distance or time*) ___________
- Medicine ___________
- Hospital stay ___________
- Productivity gains or losses (should be excluded if they are not random across disease or intervention) ___________
- Other _________

m) Pneumonia or other health-activity related impacts –

- Research related (specify) __________
- Policy Impact (specify) __________
- Services (specify) __________
- Societal (specify) _________
- Other (specify) _________

5. Identifying details mentioned:

|  | #1 | #2 (add columns as reqd.) |
| --- | --- | --- |
| 1. Individual name(s) |  |  |
| 1. Individual profession(s) |  |  |
| 1. Institution(s) |  |  |
| 1. Country(ies) |  |  |
| 1. Publication(s) |  |  |
| 1. Date(s) |  |  |

**6. Narrative Framework:**

a. Story tone-

- Negative/Tragic ________
- Positive/Triumph ________
- Neutral __________
- Other ___________

b. Meta-story/frame (main theme)-

- Human rights/Social justice/Non-discrimination _________
- Moral (Right thing to do) __________
- Global health movement/Social mobilization __________
- Economic __________
- Political/Ideological___________
- Cultural ____________
- Policy/program/plan of action __________
- Personal/heroic_____________
- Other ____________

c. Sub-themes: Check all that apply

- Human rights/Social justice/Non-discrimination _________
- Moral (Right thing to do) __________
- Global health movement/Social mobilization __________
- Economic __________
- Political/Ideological___________
- Cultural ____________
- Policy/program/plan of action __________
- Other ____________

d. Characters/Protagonists-

- Celebrity ____________ (Name _________)
- Patient/afflicted victim __________
- Healer (doctor/nurse) ___________
- Activist ____________ (Name__________)
- Organization (eg, Doctors without Borders)____________ (Name_________)
- Other ________________

e. Characters/Antagonists (Barriers)-

- Individual ___________ (Name ___________)
- Government ____________ (Name ___________)
- Organization ____________ (Name___________)
- Other ______________

f. Story dimensions (check if mentioned)-

- Broad statistics/Data __________
- Root causes / etiology___________
- Biologic agent __________
- Atypical (portrayed as exotica – e.g. affects people far away – relatively rare)__________
- Typical (quotidian problem – e.g. affects us - happens all the time)__________
- Local community context __________
- National context _________
- Global context __________
- Other __________

g. Story action-

- New or innovative initiative _____________ (Name __________)
- Other (e.g. protest, trial) ___________ (Name__________)

h. Resolution-

- Solution likely ___________ (Name __________)
- Solution not likely __________

i. Audience-

- Risk to audience (e.g., multi-drug resistant TB spread) _________ (Yes/No)
- Plea to audience ____________ (Yes/No)
  - Implicit ___________ (e.g., if the developing world funded an additional÷)
  - Explicit ___________

**7. Coders’ notes/ additional issues/catchphrases in article:**
